# Hyperspectral Imaging with Active Illumination: A Theoretical Study on the Use of Incandescent Lamp and Variable Filament Temperature

**DOI:** 10.3390/s23239326

**Published:** 2023-11-22

**Authors:** Urban Simoncic, Matija Milanic

**Affiliations:** 1 Faculty of Mathematics and Physics, University of Ljubljana, 1000 Ljubljana, Slovenia; urban.simoncic@fmf.uni-lj.si; 2 Jozef Stefan Institute, 1000 Ljubljana, Slovenia

**Keywords:** hyperspectral imaging, spectroscopy, active illumination

## Abstract

In this study, we introduce a novel hyperspectral imaging approach that leverages variable filament temperature incandescent lamps for active illumination, coupled with multi-channel image acquisition, and provide a comprehensive characterization of the approach. Our methodology simulates the imaging process, encompassing spectral illumination ranging from 400 to 700 nm at varying filament temperatures, multi-channel image capture, and hyperspectral image reconstruction. We present an algorithm for spectrum reconstruction, addressing the inherent challenges of this ill-posed inverse problem. Through a rigorous sensitivity analysis, we assess the impact of various acquisition parameters on the accuracy of reconstructed spectra, including noise levels, temperature steps, filament temperature range, illumination spectral uncertainties, spectral step sizes in reconstructed spectra, and the number of detected spectral channels. Our simulation results demonstrate the successful reconstruction of most spectra, with Root Mean Squared Errors (RMSE) below 5%, reaching as low as 0.1% for specific cases such as black color. Notably, illumination spectrum accuracy emerges as a critical factor influencing reconstruction quality, with flat spectra exhibiting higher accuracy than complex ones. Ultimately, our study establishes the theoretical grounds of this innovative hyperspectral approach and identifies optimal acquisition parameters, setting the stage for future practical implementations.

## 1. Introduction

Hyperspectral imaging has emerged as a versatile and powerful technique that enables capturing spectral information across various applications. This technology allows for the acquisition of images in numerous narrow and contiguous spectral bands, facilitating detailed analysis and characterization of materials, objects, and scenes [1]. The wealth of spectral data obtained through hyperspectral imaging has applications in remote sensing, agriculture, geology, biology, and industrial inspection.

Various hyperspectral imaging techniques have been developed to cater to different requirements and scenarios. These techniques encompass push-broom [2] and whisk-broom systems for spatial scanning, snapshot methods utilizing filters or dispersive elements, and Fourier transform-based approaches for spectral decomposition [3]. Each technique has advantages and limitations, often tailored to specific applications and equipment constraints.

Active illumination hyperspectral imaging is a specialized technique that uses controlled and deliberate illumination sources to enhance the accuracy and quality of spectral information captured in an image [4,5,6,7]. In traditional passive hyperspectral imaging, the scene is illuminated by ambient light or natural light sources, and the sensor measures the reflected or emitted light across multiple spectral bands. On the other hand, active illumination techniques introduce an element of control over the illumination source, allowing for precise manipulation of its spectral characteristics.

In active illumination hyperspectral imaging, the illumination source is typically chosen to emit light at specific wavelengths. This illumination enables the acquisition of images with more accurate spectral information, which can be crucial in applications where distinguishing between closely related materials is essential. By using controlled illumination, the signal received by the sensor can be optimized for the specific spectral bands of interest [7], which leads to improved SNR in those bands, enhancing the overall quality of the hyperspectral data. Active illumination also minimizes the impact of lighting variations due to natural lighting conditions by providing consistent and controlled lighting conditions. In addition, active illumination allows the adaptation of illumination to specific applications and compensation for sensor characteristics.

In this article, we present a different variant of active illumination hyperspectral imaging, where a spectral shape variation of broadband illumination is utilized instead of multiple spectral bands at specific wavelengths. From the images recorded with many different illumination spectra, a spectral response consisting of a small number of spectral channels is reconstructed. The light source with variable illumination spectra is an incandescent lamp with adjustable filament temperature. Control of the filament temperature can be achieved by controlling the electric current through the bulb. As the temperature of the filament changes, the emitted spectrum follows Planck’s Law for black body radiation, allowing for controlled spectral variations. In this way, the illumination spectrum is tunable with arbitrary precision, with a small caveat that the maximal filament temperature limits the available spectra variations.

The reflected or transmitted spectrum reconstruction is formulated as an inverse problem. As the illumination spectra are of similar spectral shape due to blackbody emission characteristics, the inverse problem is ill-posed, necessitating robust mathematical algorithms and regularization techniques to mitigate the ill-posedness. We present a mathematical formulation of the reconstruction algorithm and the simulation study assessing the performance of the hyperspectral imaging approach under various circumstances. Through this investigation, we seek to provide a deep understanding of the approach’s capabilities, limitations, and sensitivity to imaging parameters.

The proposed active illumination approach holds promise in scenarios where illumination is already implemented with incandescent lamps, like classical microscopy or macroscopic imaging using RGB sensors. Moreover, the simplicity and affordability of incandescent lamps make this technique accessible and practical for various novel imaging setups.

## 2. Theoretical Background

The principle of hyperspectral imaging is the recovery of an object’s spectral response for each image element. The main idea of the novel approach is to illuminate a sample with *M* known and linearly independent broadband spectra from an incandescent lamp at *M* different lamp filament temperatures; record *M* images with a camera that has *p* spectral channels with known spectral response; and reconstruct hyperspectral image with *N* < *pM* channels.

### 2.1. Incandescent Lamp Spectra

The spectrum of light emitted by an incandescent lamp changes as the temperature of the filament is varied. The Planck’s Law determines the spectrum of light emitted by the filament for black body radiation [8]:

(1) $$I\left(\lambda \right)=\frac{8\pi \mathrm{hc}}{\lambda^{5}\;}\frac{1}{e^{\frac{\mathrm{hc}}{\lambda kT}}-1}\;$$

Here, I(λ) is the spectral radiance, *h* is the Planck constant, *c* is the speed of light, *k* is the Boltzmann constant, and *T* is the temperature of the black body. The glass bulb of an incandescent lamp can also affect the spectrum of light emitted. The glass can act as a filter, absorbing specific wavelengths more than the others [9]. The absorption can change the overall spectrum of the light emitted by the bulb. The temperature of the filament in an incandescent lamp can be controlled by varying the electric current passing through it. As the current increases, so does the filament’s temperature, affecting the spectrum of light emitted.

### 2.2. Monochrome Image Acquisition

In the following text, we assume a reflectance imaging (algorithm would be the same for transmitive imaging, except that reflectance would be replaced with transmittance) and a sample reflectance *r*(*λ*). We further assume to have a sample illuminated with a light of *I*(*λ*) spectral distribution and a monochrome camera with known spectral sensitivity *ϵ*(*λ*). Here, *ϵ*(*λ*) also includes spectral dependence of imaging lens transmissivity. Under these assumptions, we obtain an imaging signal *S*:

(2) $$S=\alpha \overset{\infty }{\underset{-\infty }{\int }}I\left(\lambda \right)\epsilon \left(\lambda \right)r\left(\lambda \right)d\lambda +\eta \;$$

We have an additional scaling factor *α* that includes other factors related to data acquisition (e.g., amplification factor in acquisition electronics) and the term *η* that stands for the noise. The scaling factor can be removed by the normalization and will be omitted in the following text. Assume we have *M* illumination realizations consisting of *M* linearly independent discrete spectral distributions discretized over *N* spectral channels. The illumination can be written with the illumination matrix I(λ) that has *M* rows and *N* columns: $I_{i}\left(\lambda_{j}\right);i=1,\;\ldots ,\;M,\;j=1,\;\ldots ,\;N$. The spectral sensitivity of the imaging system is also discretized over *N* spectral channels into row-vector: $\epsilon \left(\lambda \right)\to \epsilon \left(\lambda_{j}\right);j=1,\;\ldots ,\;N$. From that, we get an imaging signal column-vector that is calculated as:

(3) $$S_{i}=\overset{M}{\underset{j=1}{\sum }}I_{i}\left(\lambda_{j}\right)\epsilon \left(\lambda_{j}\right)r\left(\lambda_{j}\right)\;$$

Here, the noise term *η* is omitted from the derivations of the signal expressions since it does not affect the actual results. However, it will be included later in the numerical simulations. In matrix notation, we have the following expression for the signal:

(4) S=(I∘(u ϵ))r,

where ∘ stands for Hadamard product, ***S*** is the *M* × 1 column-vector of imaging signals, ***I*** is the *M* × *N* illumination matrix, ***U*** is the *M* × 1 column-vector consisting of ones, ***ϵ*** is 1 × *N* row-vector consisting of imaging spectral sensitivity, and ***r*** is the row-vector consisting of spectral-dependent reflectance (hyperspectral image). In this way, we got a linear system where the signal is a result of the multiplication of the reflectance vector by the system characteristics matrix:

(5) S=A r,A=I∘(u ϵ). 

The system is overdetermined if *M* > *N* and the rows of the illumination matrix ***I*** are linearly independent, i.e., we have *M* linearly independent discrete spectral distributions. Therefore, the sample reflectance ***r*** can be reconstructed by solving the linear system (5). However, the illumination spectra from the incandescent lamp at all reasonable temperatures have similar spectral shapes, so this inverse problem is ill-posed. Here, the highest temperature is limited by the melting temperature of the filament and the lowest reasonable temperature is set by requiring certain amount of illumination power in the spectral range of the detector (power increases with temperature to 4th power) and not too much power in the infrared range.

### 2.3. Multi-Channel Data Acquisition

One solution to improve reconstruction accuracy of ***r*** is to introduce multiple spectral channels of the detector. Assuming the camera has *p* spectral channels with known spectral responses $\epsilon_{1},\ldots ,\;\epsilon_{p}$, each illumination spectrum provides *p* signals, and *M* illumination spectra provide *pM* imaging signals. With *p* channels, the system matrix becomes:

(6) $$A=I\circ \left[\begin{array}{c} u\;\epsilon_{1}\\ \vdots \\ u\;\epsilon_{p} \end{array}\right].$$

***I*** is a *pM* × *N* illumination matrix with the illumination matrix repeated *p* times column-wise, ***U*** is an *M* × *1* column-vector consisting of ones, and the signal vector ***S*** is a *pN* × 1 column-vector of *p* imaging signals. The system is overdetermined if *pM* > *N*.

### 2.4. Regularization

Due to the ill-posedness of the problem, regularization is introduced in the linear system. We used two regularization strategies:

- penalization of the second norm of solution vector ***r*** (Tikhonov regularization),
- penalization of the second norm of the first derivative of solution vector ***r***.

The Tikhonov regularization is implemented by appending the identity matrix ***J*** of size *N* × *N* with suitable weight *k*_1_ to the system matrix ***A*** of the linear system and column-vector of *N* zeros to the imaging signal vector ***S***:

(7) $$A_{r1}=\left[\begin{array}{c} A\\ k_{1}\;J \end{array}\right],\;S_{r1}=\left[\begin{array}{c} S\\ 0 \end{array}\right].$$

Penalization of the second norm of the first derivative of the solution vector is implemented similarly by appending tridiagonal matrix ***D*** of size *N* × *N* with suitable weight *k*_2_ to the system matrix ***A*** of the linear system and column-vector of *N* zeros to the imaging signal vector ***S***:

(8) $$A_{r2}=\left[\begin{array}{c} A\\ k_{2}\;D \end{array}\right],\;S_{r2}=\left[\begin{array}{c} S\\ 0 \end{array}\right],\;D=\left[\begin{array}{ccc} \begin{array}{ccc} 1 & -1 & 0\\ -1 & 2 & -1 \end{array} & \cdots & \begin{array}{c} \begin{array}{cc} 0 & 0 \end{array}\\ \begin{array}{cc} 0 & 0 \end{array} \end{array}\\ \vdots & \ddots & \vdots \\ \begin{array}{ccc} 0 & 0 & 0 \end{array} & \cdots & \begin{array}{cc} -1 & 1 \end{array} \end{array}\right].\;$$

For the best reconstruction of ***r***(*λ*), both the Tikhonov regularization and the penalization of the second norm of the first derivative are implemented simultaneously:

(9) $$A_{r}=\left[\begin{array}{c} A\\ k_{1}\;J\\ k_{2}\;D \end{array}\right],\;S_{r}=\left[\begin{array}{c} S\\ 0\\ 0 \end{array}\right],\;S_{r}=A_{r}\;r.$$

## 3. Materials and Methods

In this section, we delineate the methodology employed to carry out the simulations, assess the quality of the reconstructed spectra, and conduct a comprehensive sensitivity analysis of the novel hyperspectral approach.

### 3.1. Simulation Process

The simulation process serves as the foundational step to evaluate the efficacy of the new approach. The following subsections detail the various aspects of the simulation, including the setup, parameter choices, and the reconstruction of spectral responses.

**Figure 1:**
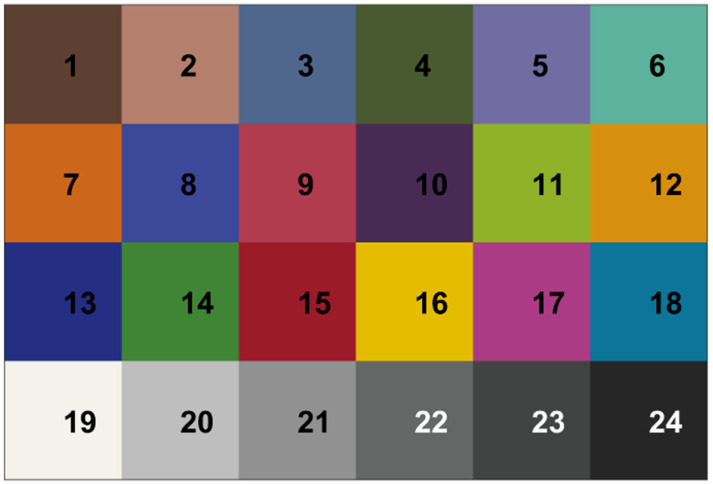
ColorChecker has the following colors and gray scales: (1) Dark skin, (2) Light skin, (3) Blue sky, (4) Foliage, (5) Blue flower, (6) Bluish green, (7) Orange, (8) Purplish blue, (9) Moderate red, (10) Purple, (11) Yellow green, (12) Orange yellow, (13) Blue, (14) Green, (15) Red, (16) Yellow, (17) Magenta, (18) Cyan, (19) White, (20) Neutral 8, (21) Neutral 6.5, (22) Neutral 5, (23) Neutral 3.5 and (24) Black. More information can be found on the manufacturer’s website (https://www.xrite.com/categories/calibration-profiling/colorchecker-classic-family/colorchecker-passport-photo-2 (accessed on 10 September 2023)).

We simulated reflectance imaging of 24 different tiles of a color standard (ColorChecker Passport 2, X-Rite, Grand Rapids, MI, USA). It is a well-established color standard and includes the 18 colored and six gray-scale tiles used as reference spectra in imaging, as each color spectrum is well-known. The colors and gray scales of the ColorChecker with the corresponding names are shown in Figure 1. The spectra of the color standard were obtained by our custom push-broom hyperspectral system [2]. A standard imaging procedure described in the reference was performed, and the spectra were calculated as the average spectra of each color tile. The spectra of each color tile served as our simulation’s simulated reflectivity *r*_s_(*λ*_i_). The spectra are presented in Results.

The chosen illumination source was incandescent light, which adheres to the principles of black body radiation (Equation (1)). The filament temperature of the incandescent lamp was systematically varied to cover a range from a minimal filament temperature of 2450 up to a 3400 K, encompassing practically achievable temperatures.

Three detector channels in the visible wavelength range were simulated: 400–500, 500–600, and 600–700 nm. We simulated these filters as top-hat filters, specifically the transmissivity of one inside the pass band and zero outside. In practice, this is achievable by applying bandpass optical filters (e.g., hard-coated filters by Thorlabs, Newton, NJ, USA).

The imaging process was modeled by assuming a CMOS monochrome imaging sensor. A realistic sensor’s quantum efficiency (data for the Sony ICX414 CMOS) was used to simulate the imaging signal acquired by the camera. The acquired images in each spectral channel were calculated according to Equation (3). Spectra were discretized with a 10 nm step, and summation was over the wavelengths that are within the selected spectral window. The imaging signal was divided by the simulated temperature to 4th power to compensate for substantially higher irradiance at higher temperatures, which would, in practice, be compensated with shorter exposure times. Calculated imaging signals were subjected to normally distributed random noise, mirroring the noise inherent in practical image acquisition scenarios.

The spectrum reconstruction process aimed to recover the original spectral reflectance of the color standard. It involved solving an overdetermined linear system (Equation (9)) and provided the estimated reflectivity ***r_e_***(*λ_i_*). Weights for the regularization were set by manual iteration until satisfactory results were obtained for each signal-to-noise ratio (SNR) value. Satisfaction with the results was determined subjectively by comparing reconstructed spectra. The same weights were used to analyze all 24 spectra and were changed only for different SNRs. The linear system was solved using Left array division function implemented in Matlab R2023a (Mathworks, CA, USA). Negative solutions were set to zero.

### 3.2. Default Simulation Parameters

The simulation was initially conducted under a set of default parameters:

- Signal-to-Noise Ratio (*SNR*): A signal-to-noise ratio of *SNR* = 100 was adopted as a typical SNR value in hyperspectral images [10].
- Filament Temperatures: A sequence of 20 different filament temperatures was selected, from 2450 to 3400 K, with Δ*T* = 50 K increments.
- No error in illumination spectrum: The filament temperature and the illumination spectrum were assumed to have no error or variation (δ*T* = 0).
- Reconstructed Spectral Step: The spectral response was reconstructed to Δ*λ* = 10 nm step.
- Number of spectral channels for image acquisition: Simulation of image acquisition assumed three spectral channels (*p* = 3) with 100 nm bandwidth, covering 400 to 700 nm range approximating RGB channels.

### 3.3. Sensitivity Analysis

The following parameters were varied to assess the approach’s sensitivity to different parameters while keeping other parameters fixed at default values:

- Signal-to-Noise Ratio (*SNR*): *SNR* values were varied to assess the algorithm’s performance under two alternative noise conditions: *SNR* = 10 and *SNR* = 1000.
- The number of Filament Temperatures: The number of filament temperatures was varied, changing temperature increments to 100 and 10 K, exploring scenarios with 10 and 100 temperature steps.
- Lowest Filament Temperature: The lowest filament temperatures were set to *T*_min_ = 1950 K and *T*_min_ = 2950 K, resulting in 30 and 10 filament temperature steps.
- Illumination spectrum Error: The algorithm’s resilience to illumination spectrum errors was examined by introducing an error in filament temperature, which had a normal distribution with a standard deviation of 10 K.
- Reconstructed Spectral Step: The spectral step of reconstructed spectra was modified to 5 and 20 nm to study the effect of higher or lower spectral resolution.
- Number of spectral channels for image acquisition: The number of simulated spectral channels *p* was set to six to assess the effect of detection channels on the reconstructed spectra, resulting in subsequential bands of 50 nm bandwidth covering the 400 to 700 nm spectral range.

### 3.4. Reconstructed Spectra Quality Assessment

For each configuration of simulation parameters and color standard spectra, the same error assessment metrics were applied to evaluate the accuracy and robustness of the approach. From the simulated reflectivity *r_s_*(*λ_i_*) and estimated reflectivity *r_e_*(*λ_i_*)*,* we calculated two error metrics:

- Root Mean Squared absolute spectral error (*RMSE*): For each reconstructed wavelength, the absolute error was calculated, squared, and averaged over the whole wavelength spectrum, and the square root was taken:

(10) $$RMSE=\sqrt{\frac{\sum_{i=1}^{N}{\left(r_{e}\left(\lambda_{i}\right)-r_{s}\left(\lambda_{i}\right)\right)}^{2}}{N}}\;,$$

- Root Mean Squared relative spectral error (*rRMSE*): For each reconstructed wavelength, the relative error was calculated, squared, and averaged over the whole wavelength spectrum, and the square root was taken:

(11) $$rRMSE=\sqrt{\frac{\sum_{i=1}^{N}{\left(\frac{r_{e}\left(\lambda_{i}\right)-r_{s}\left(\lambda_{i}\right)}{r_{s}\left(\lambda_{i}\right)}\right)}^{2}}{N}}\;.$$

### 3.5. Spectra from Real-Life Examples

To further evaluate the novel hyperspectral imaging methodology, it was simulation-tested on nine spectra from three real-life examples. The first example is microscopic hyperspectral imaging of the peritoneum of the mice colitis model. The second example is macroscopic hyperspectral imaging of an apple with mold infection. The third example is macroscopic hyperspectral imaging of basal cell carcinoma in the nose. Three spectra from each example are used for the simulation test.

## 4. Results

The signals ***S*** were simulated using the default parameters as described in the previous section. Values of regularization parameters for the default simulation were set to *k*_1_ = 10^−6^ and *k*_2_ = 10^−2^. The *k*_2_ parameter was always an inverse value of *SNR*, while *k*_1_ was for a factor of 10^4^ smaller. Suitable values for regularization parameters depend on the imaging signal’s scale.

**Figure 2:**
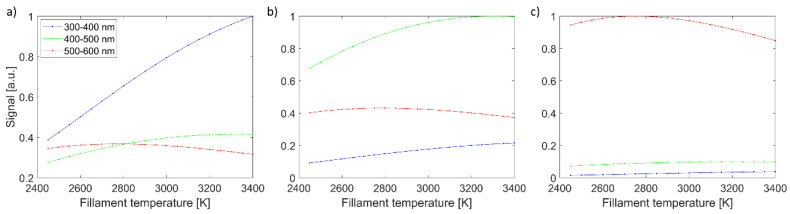
Imaging signal versus filament temperature for all three imaging channels. Three graphs are from three color spectra: (**a**) shows blue color (spectrum #13), (**b**) shows green color (spectrum #14), and (**c**) shows red color (spectrum #15). Line colors represent wavelength range.

Imaging signals for all three channels, in dependence on filament temperature, are shown in Figure 2. They are presented for selected three spectra: blue (spectrum #13), green (spectrum#14), and red (spectrum #15). As expected, the blue color (spectrum #13) with the strongest signal in the 300–400 nm channel increases with filament temperature. Similarly, the green color (spectrum #14) has the strongest signal in the 400–500 nm channel and reaches the maximum when the filament temperature is close to 3400 K. The red color (spectrum #15) is the strongest signal in the 500–600 nm channel and reaches its maximum slightly below 2800 K. All three colors have distinctive temperature dependence for three spectral channels, facilitating the reconstruction of spectra shape. It should also be stressed that the shape of imaging signal vs. filament temperature is affected by various factors, namely Planck’s black body radiation law, normalization to *T*^4^ that mimics exposition adaption to higher radiation power at higher temperature, spectral-dependant quantum efficiency of the sensor, and filter spectral dependant transmission.

**Figure 3:**
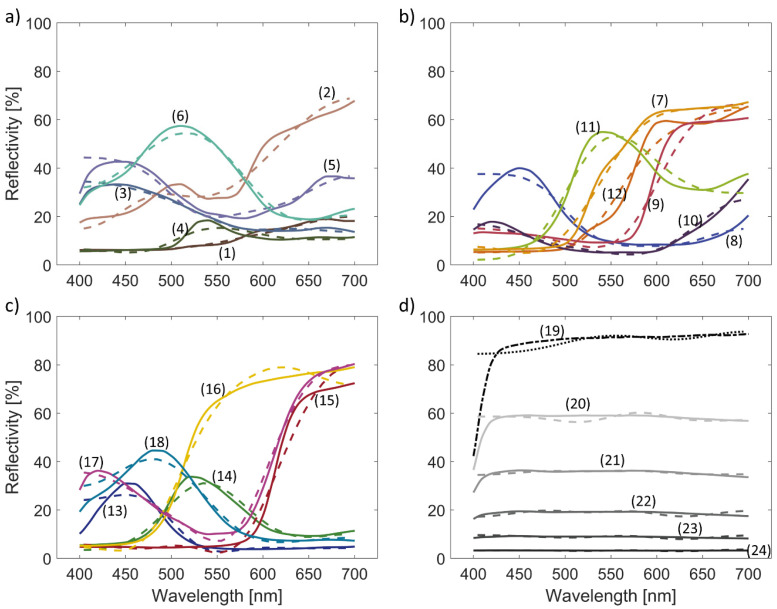
Reconstruction of reflectance spectra for all 24 colors of the color standard. The original spectra are presented in solid lines, while the reconstructed spectra are in dashed lines. Each graph represents one row of the color standard (Figure 1), namely (**a**) colors 1–6, (**b**) colors 7–12, (**c**) colors 13–18, and (**d**) colors 19–24. The colors of the graph lines correspond to the colors of the ColorChecker, except the white, which is black and dashed on the lower right.

The corresponding reconstructed spectra are shown in Figure 3. In general, the reconstructed spectra (dashed lines) follow the original spectra (solid lines), with somewhat higher errors near the edge of the spectral range in case of the spectra with increased dynamic in that spectral region (e.g., spectra #3, #5, #19). The spectra that have a transition from low to high reflectivity (e.g., spectra #16, #17, #18) typically have a well-reconstructed location of the transition. At the same time, the shape does not always fully agree with the original spectra. Some spectra featuring ripples are reconstructed with high accuracy (e.g., spectra #6, #11), while others show some discrepancies (e.g., spectra #4, #13, #16). The original grey-scale spectra are mostly flat (Figure 3d), except at the low wavelengths, where the reflectivity is significantly lower. The corresponding reconstructed spectra have some ripples and do not capture well the reduced reflectivity at low wavelengths. However, on average, the reconstructed grey-scale spectra are quantitatively close to the original spectra.

Analyzing reconstructed spectra under default simulation parameters has provided valuable insights into the algorithm’s performance. The algorithm’s ability to closely follow true spectra is evident, with slight deviations near the edges of the spectral range for some spectra. Spectra exhibiting transitions from low to high reflectivity generally exhibit accurate transition locations, although the precise shape is not always consistently captured. Notably, the spectra with ripples, such as spectra #6 and #11, demonstrate high accuracy in reconstruction. However, discrepancies arise for specific spectra like #4, #13, and #16. Original gray-scale spectra, characterized by mostly flat reflectance, show subtle ripples in the reconstructed versions. Although these reconstructions of gray-scale spectra do not fully capture the reduced reflectivity at low wavelengths, their quantitative proximity to true values is promising.

All *RMSEs* are less than 5.1%, on average less than 2.3%. The largest *RMSE* is obtained in spectrum #19, which has a reflectivity of about 90% at almost all wavelengths. For spectrum 24, which has a reflectivity of only a few percent over the entire spectrum, the error is only 0.1%. However, the results are different when we inspect the relative error *rRMSE*, considering also the amplitude of the spectra. The *rRMSE* values are generally larger, on average 9.9%, reaching 25.1% in the case of spectrum #13. Specific spectra, like spectrum #13, exhibit *RMSE* that can get up to 25%. The reason for high relative error for some spectra, like spectrum #13, is because the spectrum has ripples and relatively high error in the spectrum where the reflectivity is high, while most parts of the spectrum are low. The reconstruction errors in terms of *RMSE* and *rRMSE* are presented in Table 1.

### 4.1. Sensitivity Analysis

Changing *SNR* from 100 to 1000, which can be experimentally realized by increasing integration time or light intensity, consistently improves the *RMSE* for all reconstructed spectra. Improvement in *RMSE* is higher for some spectra (e.g., spectrum #18, where *RMSE* goes from 2.9% to 0.6%), while others have moderate improvement (e.g., spectrum #7, where *RMSE* goes from 3% to 2.5%). On average, the *RMSE* was reduced to 1.4% compared to 2.3% for *SNR* = 100. In general, *rRMSE* decreases by increasing *SNR*, but surprisingly, it also slightly increases for some spectra (spectra #7, #12. #15, and #15, where the *RMSE* does not improve considerably). The reason for such unexpected behavior of *rRMSE* is that those spectra have ripples, hence the notable error in the area of very low reflectivity in the case of *SNR* = 1000. Therefore, a substantial relative error in this part of the spectrum is present (note that the absolute error is normalized to the true value of reflectivity, which is very low) and, consequently, the increase in *rRMSE*. The increased *rRMSE* is possible because our spectrum reconstruction uses the means for least squares absolute error, so it tries to find the minimum in *RMSE*, not the *rRMSE*. The average *rRMSE* in the case of *SNR* = 1000 is still improved; it is 8.2% compared to 9.9% for *SNR* = 100, resulting in a much smaller error reduction than *RMSE*. The errors for all spectra are collected in Table 2 and Table 3.

A similar improvement as with the increased *SNR* is achieved by doubling the number of acquisition spectral channels to *p* = 6. The average *RMSE* is 1.4%, and the average *rRMSE* is 7.2%. The reconstruction error reduction is smaller in the case of the decrease in the filament temperature step (Δ*T* = 10 K). Namely, the average *RMSE* is 1.8%, and the average *rRMSE* is 8.3%. Increasing the number of acquisition spectral channels and smaller increments in filament temperature provides twice and five times more measurements, which have the same effect as improved *SNR*. When combining repeated measurements, the *SNR* increases with the square root of the number of repeated measurements. We expect similar behavior (i.e., an apparent improvement in *SNR*) if the number of acquisition spectral channels is doubled or the number of filament temperatures is five times higher. The apparent improvement in *SNR* should be for the $\sqrt{2}$ or $\sqrt{5}$. A smaller increment in filament temperature provides information that could be obtained by interpolating the data with default simulation parameters. Hence, the improvement is likely due to more data and apparent improvement of the *SNR*. Increasing the number of acquisition spectral channels provides better spectral resolution in the measured data, so improvement in reconstructed spectra is also expected due to this reason. Worse performance for *p* = 6 acquisition spectral channels, compared to *SNR* = 1000 in gray-scale spectra, is because improved *SNR* facilitates better estimation of relatively low reflectivity at low wavelength range.

In the next step, we impaired these three imaging parameters. By reducing *SNR* to 10, the reconstruction accuracy also reduces. The average *RMSE* is 3.8% compared to 2.3% for the default set, and the average *rRMSE* is 23.7% compared to 9.9% for the default set. Similarly, by increasing Δ*T* to 100 K, reconstruction accuracy is reduced, resulting in the average *RMSE* = 2.7% and *rRMSE* = 11.8%. However, the errors increase substantially less when increasing Δ*T* than decreasing *SNR*, thus showing that the reconstruction is very sensitive to low *SNR*. A more significant increment in filament temperature does not exclude the information that could not be obtained by interpolating the data with default simulation parameters, so slightly worse results are likely due to less data and apparent degradation of the *SNR*. That explains why reducing *SNR* tenfold (to *SNR* = 10) affects the accuracy of reconstructed spectra more severely than the simulated larger filament temperature increments, which would reduce the apparent *SNR* only for a factor of $\sqrt{2}$.

Then, we tested the effect of minimal filament temperature on the reconstructed spectra accuracy and found that by decreasing the minimum filament temperature, a slight improvement in the reconstruction accuracy was achieved. Still, the increase in the minimum filament temperature results in a significant reduction in the reconstruction accuracy. Specifically, if the minimum temperature is decreased to 1950 K, the average *RMSE* and *rRMSE* are 1.9% and 8.9%, respectively. If the minimum temperature increases to 2950 K, the average *RMSE* and *rRMSE* are 3.1% and 18.2%, respectively. These results can be explained by noting that lower and higher minimal filament temperatures determine how different illumination spectra are used in the inverse problem. Going to a very low filament temperature cannot have much effect because, in that case, the spectrum is heavily shifted towards infrared. Even at the maximal achievable temperature *T* = 3400 K, the black body radiation spectrum has maximum in infrared (*λ*_max_ = 853 nm). Still, this spectrum has to be weighted with the CMOS quantum efficiency, which rapidly decreases at 700 nm and above.

On the other hand, if the lowest temperature is set at a higher value, we omit spectra at lower temperatures. Consequentially, the inverse problem is formed with similar spectra, and the reconstruction problem is more ill-posed. In addition, increasing the lowest temperature also reduces the number of available temperatures and, therefore, the number of acquired signals and the apparent *SNR*. All of that explains the lower accuracy of the reconstructed spectra when the minimal temperature was raised to *T*_min_ = 2950 K.

The next test was introducing the illumination spectra errors by assuming the wrong estimation of the filament temperature (δ*T*). While *RMSE* is still below 10%, on average 4.8%, the spectra are visually inaccurate. That is especially true for the gray-scale spectra (spectra #19 to #24), where pronounced ripples appear. Poor quality of the reconstructed spectra is also revealed by the *rRMSE*, which reaches 45% and is, on average, 25.7%. These results demonstrate that the filament temperature error is the most prominent source of error in the reconstructed spectra and should not be surprising. Our hyperspectral algorithm relies on inverse reconstruction of the spectra, using linearly independent illumination spectra, but they are of similar spectral shape. As the spectra are similar, it is not surprising that the algorithm is sensitive to the error of the illumination spectra. Even with a small spectral error (δ*T* = 10 K), differences among the different temperature illumination spectra are lost due to the spectral error. Therefore, this issue must be adequately addressed.

The final sensitivity test was changing the wavelength step size of the reconstructed spectra Δ*λ*. It has some effect, but it depends on the actual spectrum. In some cases, smaller wavelength step size improves accuracy, and larger reduces it (e.g., #6, #14, or #18), while in other cases, the smaller wavelength step even reduces the *rRMSE* (e.g., #3 or #19). Specifically, when Δ*λ* is reduced to 5 nm, the average *RMSE* and *rRMSE* are 2.2% and 11.6%, respectively. When Δ*λ* increases to 20 nm, the average *RMSE* and *rRMSE* are 2.9% and 16.0%, respectively. The reasons for such behavior are not clear. However, the spectral resolution of the reconstructed spectra is significantly better than the resolution of the detected spectra (*p* = 3 or 6), implying that the subtle differences could arise from the regularization techniques or other sources.

The complete results of the sensitivity analysis are shown in Table 2 and Table 3. The reconstructed reflectance spectra, together with the original spectra for all parameter variations within the sensitivity analysis, are included in Supplementary Materials.

### 4.2. Spectra from Real-Life Examples

**Figure 4:**
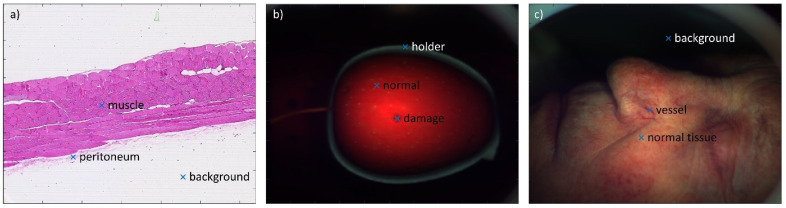
RGB images from real-life examples: (**a**) microscopic transmission hyperspectral imaging of peritoneum of the mice colitis model, (**b**) macroscopic hyperspectral imaging of apple with mold infection, and (**c**) macroscopic hyperspectral imaging of basal cell carcinoma in the nose.

RGB images from real-life examples are shown in Figure 4. Three points where the spectra were taken for the simulation tests are marked on those images.

**Figure 5:**
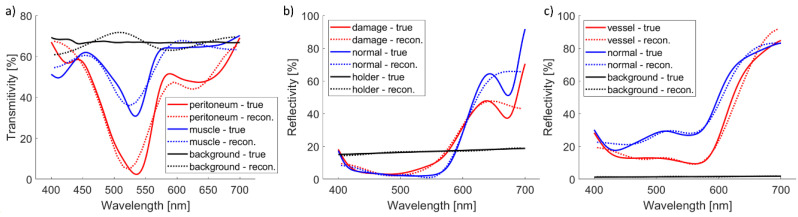
Simulated and reconstructed spectra from real-life examples: (**a**) microscopic transmission hyperspectral imaging of peritoneum of the mice colitis model, (**b**) macroscopic hyperspectral imaging of apple with mold infection, and (**c**) macroscopic hyperspectral imaging of basal cell carcinoma in the nose.

Simulated and reconstructed spectra are shown in Figure 5. Background or apple holder have flat spectra, which are correctly reconstructed. Other spectra are more complex and do not have all details properly reconstructed. However, the basic shape is reconstructed correctly.

Accuracy in terms of RMSE and rRMSE is reported in Table 4. While RMSE is always within 5%, rRMSE can be high. That is the case when part of the true spectrum is very low, and the relative spectrum error is significant even though the absolute error is not that large (e.g., peritoneum around 550 nm).

## 5. Discussion

This study presents a novel hyperspectral imaging approach that harnesses the potential of active illumination and a small number of detection channels. Our choice to explore three- and six-channel scenarios reflects the practical feasibility of integrating this approach into existing imaging setups. The three-channel configuration is particularly advantageous due to its compatibility with commonly used filter cubes in microscopes and RGB cameras. In contrast, the six-channel configuration aligns with the prevalent design of filter wheels. The findings of this study are relevant to both reflectance imaging, which was the selected imaging method in this study, and transmittance imaging since the imaging approach and spectral reconstruction algorithm are the same.

A critical result of our simulation-based investigation is the approach’s sensitivity to the accuracy of the illumination spectrum. Introducing illumination spectrum errors by assuming incorrect filament temperatures is a significant source of the reconstruction error. While *RMSE* remains generally below 10% and often below 5%, visual discrepancies are prominent, especially in the gray-scale spectra with notable ripples. The elevated *rRMSE* values, reaching up to 45%, underscore the algorithm’s sensitivity to the accuracy of illumination spectra. Therefore, we must always remember the necessity of precisely knowing the emission spectrum of the illumination source, which can be achieved through spectrometer-based measurements. For example, a fiber probe can collect the emitted spectrum, which a spectrometer samples in real-time. The accurate illumination spectrum information is a foundation for successful spectral reconstruction, ensuring that the algorithm can effectively separate and recover the unique spectral characteristics of the imaged samples.

An alternative avenue for the calibration would involve imaging spectral standards of well-defined spectra at different filament temperatures and subsequently calibrating the system using these standards. However, the effectiveness of this approach hinges on the repeatability of the incandescent lamp’s emission spectrum at these temperatures. If the spectrum remains consistent across different imaging sessions, it could provide a practical calibration route, particularly when spectrometer-based calibration is challenging.

Other parameters that were systematically varied to assess their impact on reconstruction accuracy also affect the algorithm’s accuracy. The first two parameters are the *SNR* and the number of acquisition spectral channels *p*. Elevated *SNR* from 100 to 1000 consistently improves *RMSE* across all reconstructed spectra. Prominent *RMSE* reduction is observed for some spectra (e.g., #18) and only moderate improvements for others (e.g., #7). Interestingly, the *rRMSE* findings differ slightly, with improved *SNR* generally leading to reduced *rRMSE*, though occasional increases occur (e.g., #7 and #12). Investigation of the reconstructed spectra shows that the Orange for the *SNR* = 1000 has a higher error in the part with very low reflectivity, which results in substantial relative error for that part of the spectrum. The effectiveness of improving reconstruction accuracy through an increased number of acquisition spectral channels (up to *p* = 6) or a smaller increment in filament temperature (Δ*T* = 10 K) is evident. While both strategies enhance accuracy, the impact of increased spectral channels is more pronounced, underscoring the importance of data quantity and quality in hyperspectral reconstruction. Decreasing *SNR* to 10 consistently degrades reconstructed spectra accuracy. However, the magnitude of reduction varies among different colors and gray-scale spectra. Particularly, *rRMSE* values are more adversely affected than *RMSE*, emphasizing the algorithm’s sensitivity to relative errors under low *SNR* conditions. Similar trends emerge with larger filament temperature increments (Δ*T* = 100 K), albeit with less pronounced effects. Notably, specific spectra, like #6, perform relatively well with larger increments in filament temperature but exhibit poorer results with lower *SNR*.

The influence of filament temperature range selection on reconstruction accuracy is also explored. Lower minimal filament temperature has a marginal effect on accuracy, while higher minimal temperature leads to reduced accuracy. This outcome highlights the role of illumination spectra diversity in the inverse problem, particularly in scenarios where similar spectra exacerbate ill-posedness. The analysis of varying wavelength step sizes in reconstructed spectra demonstrates mixed outcomes. Smaller step sizes enhance accuracy in some cases but worsen it in others, a pattern also observed with larger step sizes.

Our simulations involved spectral channels with sharp passbands commercially available at optical component sellers like Thorlabs. While these filters introduce small ripples within the passband, it is reasonable to assume that they are unlikely to significantly impact the algorithm performance because they are often on a wavelength scale lower than the spectral resolution of this hyperspectral approach. However, it is essential to validate this aspect during the actual implementation.

The simulation study leaned on the regularization techniques for reflectivity spectra reconstruction. Acknowledging that these regularization techniques may not be universally optimal across all scenarios is pertinent. The potential for enhancing results by optimizing these techniques underscores the iterative nature of algorithm refinement and the scope for further improvements in accuracy and applicability. Like the regularization optimization, optimizing the illumination protocol is also essential to implementing the method. Higher filament temperature increases the radiation power (proportional to *T*^4^) and spectrum. Increased radiation power should be compensated by decreased exposure time to prevent under- or overexposure. We simulated that by *T*^−4^ normalization of the illumination spectra from Planck’s law (Equation (1)). However, different normalizations might improve spectrum identifiability as the illumination power in pass wavelength band is not proportional to *T*^4^.

This study partially relates to existing active illumination spectral imaging [4,5,6,7]. However, it fundamentally differs from these studies by introducing continuously variable spectrum illumination, possibly enabling the reconstruction of far more spectral points than active illumination using a few distinct spectra. Specifically, Kaariainen and Donsberg combined a supercontinuum laser (SC) and a Fabry–Perot interferometer (FPI) to build a tunable light source enabling active illumination at selected spectral bands. Since the spectral transmittance of FPI is tuned by voltage, custom spectral bands can be selected, resulting in multiple spectral images (100 in this study). In contrast to our approach, a set of monochromatic images is recorded in [4], like the filtered hyperspectral imaging method [11]. However, the SC–FPI active illumination hardware is expensive and bulky compared to our approach and cannot be easily implemented in existing imaging setups. A similar method of active illumination for HIS was implemented in [5]. Here, 27 LEDs with different and disjunct spectral emissions covering the 300–1050 nm spectral region were combined in a light-pipe module carefully designed to provide a homogenous illumination field. The same illumination idea, but in a ring illumination geometry, was demonstrated also in [7]. In this study, 19 LEDs covering the spectral range 365–1050 nm were distributed within a ring. These two approaches are as expensive as the SC-FPI illumination. The imaging is limited to only a small number of preselected spectral bands, while the illumination modules are still bulky and mostly incompatible with existing imaging setups. The authors of these studies showed some HSI results, namely spectra and spatial images, but they neither provided a comparison to reference imaging modalities nor assessed the effect of different imaging parameters on the imaging performance. However, it is necessary to point out that our proposed active illumination differs entirely from the above methods since multiple images integrated over the whole detection spectral range are acquired, and hypercubes are obtained by performing reconstruction.

Traditionally, the approach with active illumination is to perform imaging of multiple narrow spectral bands [4,5,6,7]. There are also methods for spectra reconstruction from an RGB image, which is an underdetermined problem and cannot be solved without a-priori information [12]. Moreover, it is argued that the same RGB can map to different spectra depending on the context [13]. The problem is typically solved by deep neural networks (DNN). One DNN approach primarily focuses on training a “pixel-centric” mapping, wherein each pixel’s RGB values are mapped to its spectral estimate without considering neighboring pixels [12,13,14,15]. More recently, DNN has shifted towards “patch-centric” mappings. In this approach, substantial image content information is anticipated to be extracted from extensive image patches and integrated into the super-resolution process [16,17]. However, our proposed approach does not need a-priori information since multiple spectral images are recorded, which are used to reconstruct hyperspectral cubes.

Like the spectrum reconstruction from RGB images, our approach to hyperspectral imaging could produce better results if the regularized inversion of the mathematically formulated problem was inverted with machine learning techniques such as Random Forest, Artificial Neural Networks (ANN), and Convolutional Neural Networks (CNN). Improved results are anticipated due to the inherent nonlinearity of machine learning methods, which should yield superior results.

The inherent problem of our approach is the spectrum of the incandescent lamp, which has substantial power in red and NIR ranges for all achievable filament temperatures. This problem could be solved with a custom-designed filter that has high transmission in the blue region and gradually decreased transmission for longer wavelengths. However, an appropriate filter may not be available as a standard component, so it should be custom-designed, which likely defeats the idea of having a simple and cheap hyperspectral imaging system. On the other hand, that might not be a problem if the presented concept of hyperspectral imaging is employed in a device that is produced in a large quantity.

Given their widespread availability and simplified hardware requirements, the allure of employing RGB cameras instead of dedicated spectral filters is evident. However, this transition poses notable challenges. Spectral bands in RGB cameras tend to overlap, which could affect the separability of the spectral information. Furthermore, some RGB cameras exhibit outstanding quantum efficiency in the red and near-infrared (NIR) regions for all three spectral channels. Therefore, successfully adapting this methodology to RGB cameras necessitates meticulous camera selection, focusing on cameras with low quantum efficiency for the G and B channels within the red and NIR wavelength ranges or using a suitable cut-on optical filter to eliminate the NIR light contribution to the signal.

The simulation study primarily explored hyperspectral imaging within the optical 400–700 nm range, leveraging the standard ColorChecker spectra. However, we posit that the technique’s potential extends to wider spectral ranges. The CMOS cameras can operate within the 300–1000 nm range, which presents new opportunities for hyperspectral imaging in this wavelength range, but they have lower quantum efficiency in the UV and NIR range. However, we must note the interplay between the camera’s quantum efficiency and the incandescent lamp’s emission spectrum within the NIR range. This compensatory effect should contribute to the algorithm’s viability in this extended range.

We have discussed some of the study’s limitations earlier (e.g., ignoring the actual transmissivity of the bandpass filters that may have ripples). Another limitation was using a direct method to solve linear systems and obtain reflectance spectra from the simulated signals. Iterative methods may yield better results, such as preconditioned conjugate gradients or least squares. We used a simple *T*_4_ normalization factor to normalize the signals at different temperatures. However, this may not be the best approach, and we should explore other normalization strategies. We used the same regularization weights, *k*_1_ and *k*_2*,*_ for the same SNR values. Yet, the optimal weights may vary depending on the signal shapes and noise levels. We could develop a method to select the optimal weights based on these factors. We used ColorChecker as a sample to evaluate our approach. ColorChecker is a standard reference object in visible light imaging, RGB, and spectral imaging, but we could also simulate other reference standards, such as skin colors or minerals, to mimic specific applications. We expect that using more suitable reference standards would improve the reconstructed spectra for these narrower sample sets. Finally, the main limitation of this study is that we only report numerical simulation results without experimental validation. However, this study aims to suggest the possibility of using active illumination for hyperspectral imaging, which needs to be tested experimentally in the future.

For implementation of this technique in real-world applications, some challenges remain to be solved. One of them is the accurate determination of illumination spectra, which could be solved by including a spectrometer in the system to measure the spectrum of the emitted light in real-time. Proper calibration makes it possible to provide the spectrum of adequate accuracy as the input parameter of the reconstruction technique. The information about the spectral sensitivity of the detector, including the filters’ transmissivity, can be obtained either from the manufacturer or experimentally measured. Another possible solution to get an accurate transformation matrix ***A*** is to thoroughly calibrate different measured illumination spectra on different reflectance standards. This information is used to estimate the ***A***’s for different imaging conditions by the Wiener estimation method [18]. In this case, it is unnecessary to know the detector sensitivity and filter transmissivity since they would already be part of the estimated Wiener transformation matrices. Another challenge is the time needed to change the incandescent bulb filament temperature. The filament temperature is approximately proportional to the electric current; thus, it is possible to change the filament temperature rapidly by rapidly changing the current. It was shown that under normal operating conditions, starting at 300 K after turning on an incandescent lamp, it reaches its final temperature of approx. 3000 K in less than 0.5 s [19]. Based on that, a quick estimate would be that to change filament temperature to 50 K, it would be necessary to wait 10 ms. A complete imaging involving 20 temperature steps would take less than 1 s. In real imaging conditions, this time would be longer due to the image recording, acquisition of spectra, and other steps involved in the imaging procedure, but the imaging would be relatively fast. A challenge might also be integrating the new imaging components, including the programmable current source and spectrometer, in an existing system. However, in most imaging systems, this should not be a problem since both components are relatively small, compact, and non-expensive.

The versatility of this approach is underscored by its applicability to both hyperspectral microscopy and macroscopic hyperspectral imaging. However, the design of illumination schemes and calibration procedures must be tailored to the specifics of each technique. Integrating this approach into hyperspectral microscopy could revolutionize the spatial analysis of biological samples, while its application to macroscopic imaging holds promise in diverse fields such as medicine, material characterization, and industrial inspections.

## 6. Conclusions

In conclusion, this study’s novel hyperspectral approach marks a significant advancement in hyperspectral imaging. We demonstrate the feasibility of integrating active illumination using a variable filament temperature incandescent lamp and a small number of acquisition channels. Our findings show the applicability of the approach for hyperspectral imaging. However, they also underscore the importance of knowing the accurate illumination spectrum and highlight other potential challenges in the practical implementation. With its simplicity and broad applicability, it shows a potential to provide new insights across various domains where spectral information is of importance.

## Figures and Tables

**Table 1:** Absolute (*RMSE*) and relative Root Mean Square Error (*rRMSE*) in (%). The positions in the table correspond to the positions of color tiles on the color standard (Figure 1).

*RMSE (%)* *(specter number)*	1.0 (#1)	2.8 (#2)	1.3 (#3)	1.2 (#4)	2.3 (#5)	1.3 (#6)
	3.0 (#7)	3.2 (#8)	3.5 (#9)	1.5 (#10)	3.5 (#11)	1.8 (#12)
	3.3 (#13)	1.8 (#14)	3.7 (#15)	2.6 (#16)	2.6 (#17)	2.9 (#18)
	5.1 (#19)	3.8 (#20)	1.1 (#21)	1.0 (#22)	0.5 (#23)	0.1 (#24)
*rRMSE (%)* *(specter number)*	7.5 (#1)	7.3 (#2)	6.1 (#3)	13.4 (#4)	8.0 (#5)	4.8 (#6)
	9.6 (#7)	13.8 (#8)	9.7 (#9)	8.6 (#10)	13.7 (#11)	11.8 (#12)
	25.1 (#13)	9.7 (#14)	20.9 (#15)	12.1 (#16)	9.2 (#17)	11.5 (#18)
	9.3 (#19)	8.2 (#20)	3.3 (#21)	5.5 (#22)	5.8 (#23)	2.8 (#24)

**Table 2:** The *RMSE* in reconstructed spectra (%) for perturbated simulation parameters.

Modified Parameter	Default Parameters	*SNR*	*SNR*	Δ*T*	Δ*T*	*T*_min_	*T*_min_	δ*T*	Δ*λ*	Δ*λ*	*p*
New Value		1000	10	10 K	100 K	1950 K	2950 K	10	5 nm	20 nm	6
Dark skin	1.0	0.4	0.8	0.5	0.7	0.6	0.5	2.9	0.8	0.4	0.4
Light skin	2.8	2.0	4.5	2.1	4.8	2.7	4.3	7.5	3.9	3.2	2.0
Blue sky	1.3	0.8	2.6	1.3	1.9	1.2	1.2	3.7	1.7	1.5	0.8
Foliage	1.2	1.0	2.3	1.1	1.2	1.2	1.9	3.4	1.4	1.4	0.9
Blue flower	2.3	1.5	3.4	1.5	3.0	1.8	3.6	3.2	2.4	2.4	1.4
Bluish green	1.3	0.7	5.4	0.8	2.0	1.0	4.2	4.5	0.9	4.0	0.7
Orange	3.0	2.5	5.5	2.5	2.7	2.9	4.6	5.5	3.4	4.1	2.5
Purplish blue	3.2	2.1	3.8	2.2	3.4	2.6	3.2	2.5	2.2	4.0	1.9
Moderate red	3.5	1.9	5.9	2.6	5.2	2.3	5.6	7.7	4.2	5.2	1.8
Purple	1.5	1.0	2.9	1.1	1.8	1.1	2.1	3.3	1.6	1.9	1.1
Yellow green	3.5	1.2	5.8	2.2	3.0	2.4	5.5	5.8	2.8	5.4	1.0
Orange yellow	1.8	1.4	5.0	2.4	2.4	2.0	2.0	5.1	1.7	3.2	1.4
Blue	3.3	2.4	4.7	2.5	3.8	2.9	4.6	2.6	2.5	4.6	2.2
Green	1.8	0.8	4.6	1.1	1.6	1.5	3.2	5.2	1.0	3.0	0.7
Red	3.7	2.4	5.1	2.5	3.7	2.8	3.8	8.1	2.5	3.8	2.2
Yellow	2.6	1.6	4.2	2.5	5.4	1.6	3.3	5.6	3.5	3.5	1.4
Magenta	2.6	1.8	3.7	2.2	4.1	3.1	3.0	8.5	2.2	3.0	1.7
Cyan	2.9	0.6	7.6	1.0	3.3	1.2	4.4	3.3	0.9	5.1	0.6
White	5.1	4.6	6.4	5.5	5.2	5.5	5.2	7.3	6.7	5.2	4.9
Neutral 8	3.8	2.3	3.6	2.8	3.0	2.8	2.9	7.0	3.5	2.2	2.2
Neutral 6.5	1.1	0.8	1.6	1.1	1.3	1.4	1.1	4.9	1.2	0.7	0.9
Neutral 5	1.0	0.3	0.6	0.4	0.9	0.5	2.3	3.7	0.7	0.7	0.3
Neutral 3.5	0.5	0.1	0.7	0.3	0.9	0.2	0.8	2.4	0.8	0.2	0.1
Black	0.1	0.0	0.4	0.0	0.1	0.1	0.5	1.4	0.3	0.2	0.1

**Table 3:** The *rRMSE* in reconstructed spectra (%) for perturbated simulation parameters.

Modified Parameter	Default Parameters	*SNR*	*SNR*	Δ*T*	Δ*T*	*T*_min_	*T*_min_	δ*T*	Δ*λ*	Δ*λ*	*p*
New Value		1000	10	10 K	100 K	1950 K	2950 K	10	5 nm	20 nm	6
Dark skin	7.5	3.7	8.5	4.6	8.2	4.3	4.4	27.9	6.2	3.3	3.1
Light skin	7.3	5.2	17.7	6.1	10.3	7.4	17.0	19.6	7.7	11.0	5.1
Blue sky	6.1	3.2	9.6	5.0	7.6	6.1	4.4	22.4	8.2	5.4	3.3
Foliage	13.4	12.1	26.0	12.1	11.2	11.2	26.0	32.2	19.9	14.4	11.3
Blue flower	8.0	4.6	10.4	4.6	9.3	5.6	10.7	10.0	7.3	6.7	4.1
Bluish green	4.8	2.3	18.0	2.7	5.6	3.0	14.3	15.8	3.1	14.2	2.3
Orange	9.6	12.5	29.2	8.9	8.0	8.9	16.9	28.3	16.4	15.7	7.7
Purplish blue	13.8	9.3	17.5	9.5	14.9	11.0	16.4	18.1	12.0	16.8	8.7
Moderate red	9.7	8.2	21.7	8.9	14.0	11.7	20.5	31.2	10.5	18.8	7.0
Purple	8.6	5.1	22.3	5.9	11.3	5.5	13.1	29.7	13.1	12.4	4.9
Yellow green	13.7	11.2	40.5	8.6	21.2	9.4	31.6	37.8	11.9	38.6	9.0
Orange yellow	11.8	12.5	54.3	13.7	18.1	13.9	19.2	18.4	9.8	33.0	11.9
Blue	25.1	20.1	36.3	21.8	28.5	22.6	35.8	33.0	23.4	31.5	17.3
Green	9.7	5.5	42.6	5.6	12.1	14.9	37.4	44.3	5.7	29.9	5.0
Red	20.9	28.8	68.0	27.4	26.7	24.7	39.4	33.2	33.9	32.2	23.3
Yellow	12.1	19.0	43.5	13.7	16.9	12.4	42.4	33.3	28.3	40.3	14.7
Magenta	9.2	9.7	21.3	7.6	9.1	7.9	11.4	19.4	11.5	13.1	8.8
Cyan	11.5	2.9	34.3	5.8	12.9	7.0	17.8	32.4	5.6	19.7	3.3
White	9.3	8.0	11.4	9.2	9.2	9.6	8.6	8.7	10.6	8.1	8.8
Neutral 8	8.2	5.0	7.9	5.9	6.7	6.0	6.5	12.2	6.8	4.5	4.8
Neutral 6.5	3.3	2.4	4.9	3.5	4.1	4.0	3.6	14.1	3.9	2.1	2.9
Neutral 5	5.5	1.6	3.5	2.3	5.0	3.0	12.3	20.5	4.0	3.7	1.8
Neutral 3.5	5.8	1.6	7.9	3.9	10.0	2.3	9.3	28.2	9.0	2.8	1.7
Black	2.8	1.5	12.5	1.4	3.2	2.2	17.2	44.8	8.6	5.6	1.6

**Table 4:** The RMSE and *rRMSE* of reconstructed spectra from real-life examples (%).

	*RMSE*			*rRMSE*		
	(a)	(b)	(c)	(a)	(b)	(c)
Red graph	4.2	4.4	3.7	50.8	25.0	9.2
Blue graph	3.2	4.9	2.4	6.9	23.4	7.8
Black graph	3.5	0.4	0.1	5.1	2.4	8.0

## Data Availability

The data that support the findings of this study are available upon reasonable request from the authors.

## Supplementary Materials

The following supporting information can be downloaded at: https://www.mdpi.com/article/10.3390/s23239326/s1, Figure S1: Reconstructed reflectivity spectra for SNR = 1000; Figure S2: Reconstructed reflectivity spectra for SNR = 10; Figure S3: Reconstructed reflectivity spectra for ΔT = 10 K; Figure S4: Reconstructed reflectivity spectra for ΔT = 100 K; Figure S5: Reconstructed reflectivity spectra for Tmin = 1950 K; Figure S6: Reconstructed reflectivity spectra for Tmax = 2950 K; Figure S7: Reconstructed reflectivity spectra for δT = 10 K; Figure S8: Reconstructed reflectivity spectra for Δλ = 5 nm; Figure S9: Reconstructed reflectivity spectra for Δλ = 20 nm; Figure S10: Reconstructed reflectivity spectra for *p* = 6 acquisition channels.
