# Supplementary material for: Hyperspectral Imaging with Active Illumination: A Theoretical Study on the Use of Incandescent Lamp and Variable Filament Temperature
Source: Sensors (Basel). 2023 Nov 22;23(23):9326. doi: 10.3390/s23239326 (PMC10708585; doi:10.3390/s23239326)
Supplement: Supplementary file 1 [file sensors-23-09326-s001.zip › sensors-2647857-supplementary.pdf]

Reconstructed reflectivity spectra, together with original spectra for all parameter variations within the sensitivity analysis

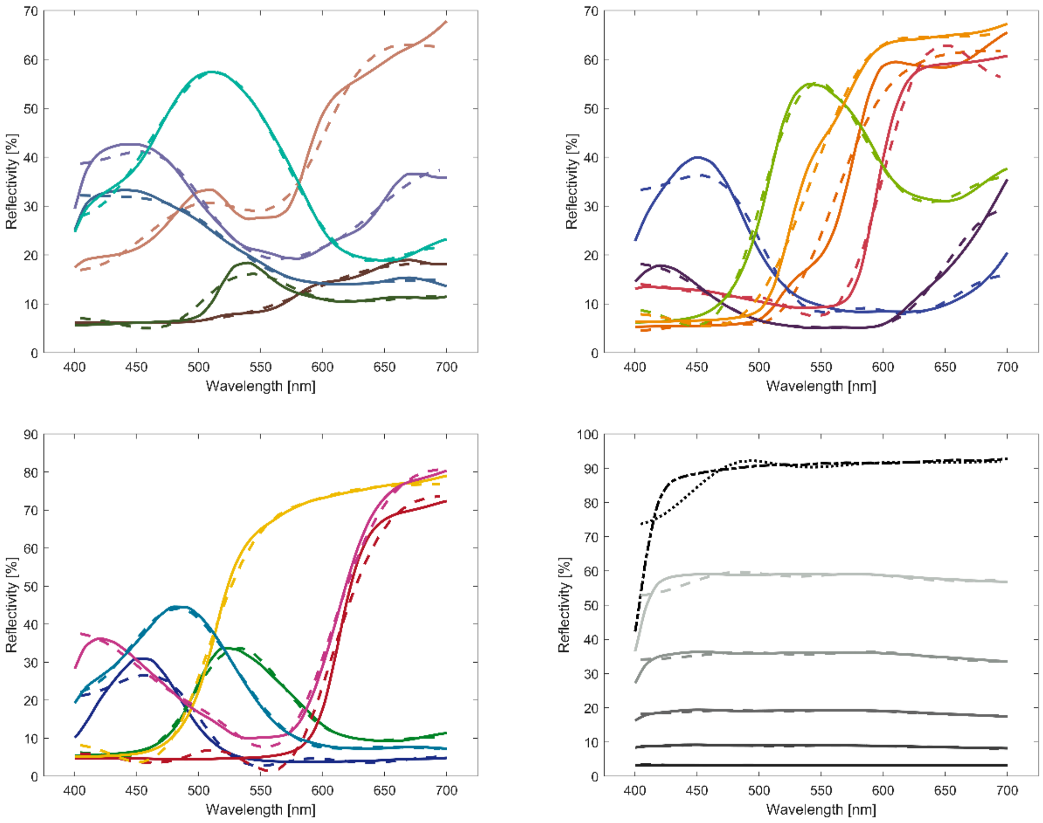


*Figure 1: Reconstructed reflectivity spectra for **SNR=1000**.*

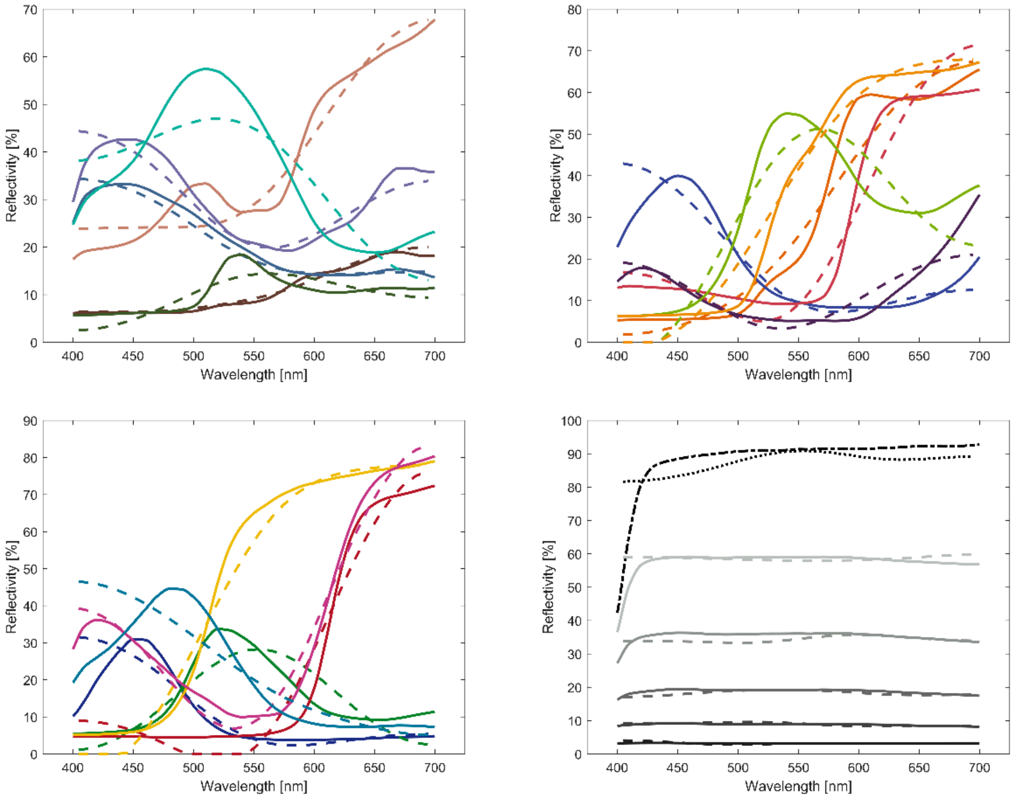


*Figure 2: Reconstructed reflectivity spectra for* ***SNR=10****.*

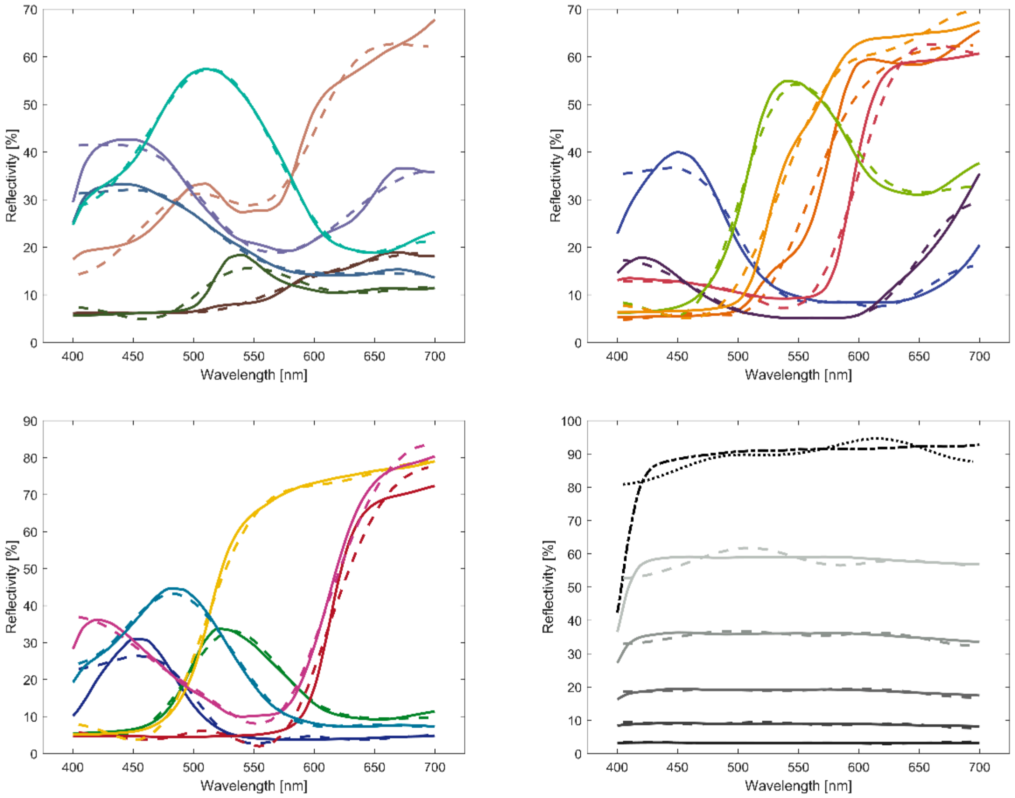


*Figure 3: Reconstructed reflectivity spectra for* ***ΔT=10K****.*

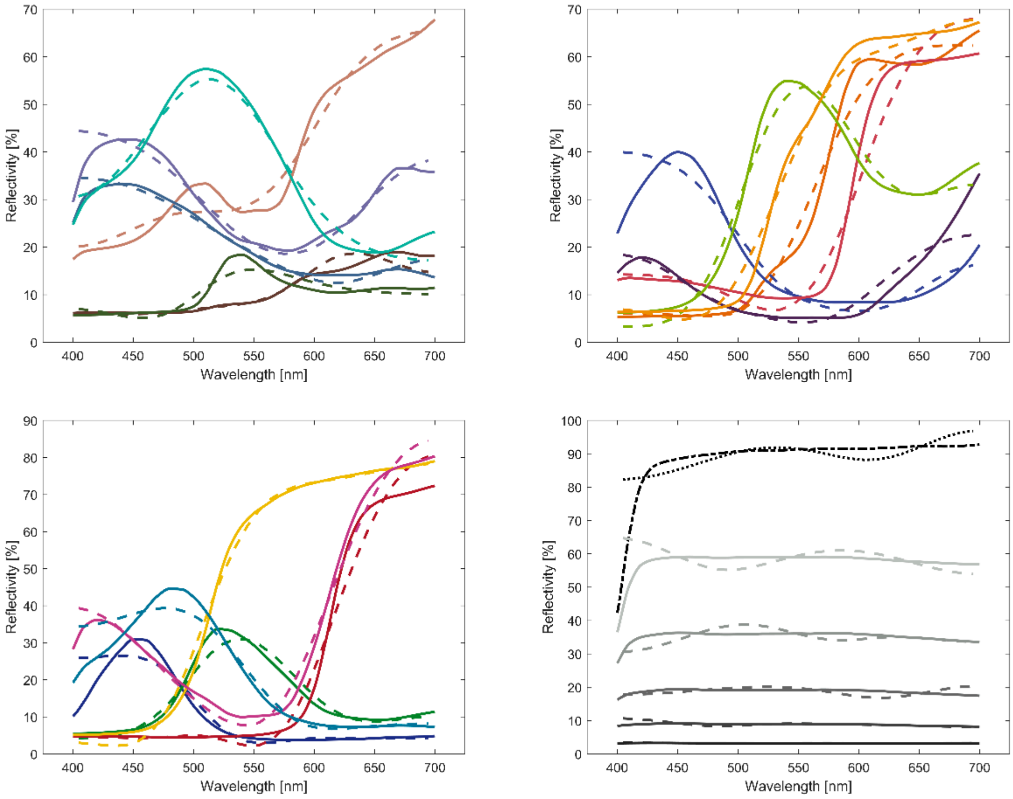


*Figure 4: Reconstructed reflectivity spectra for* ***ΔT=100K****.*

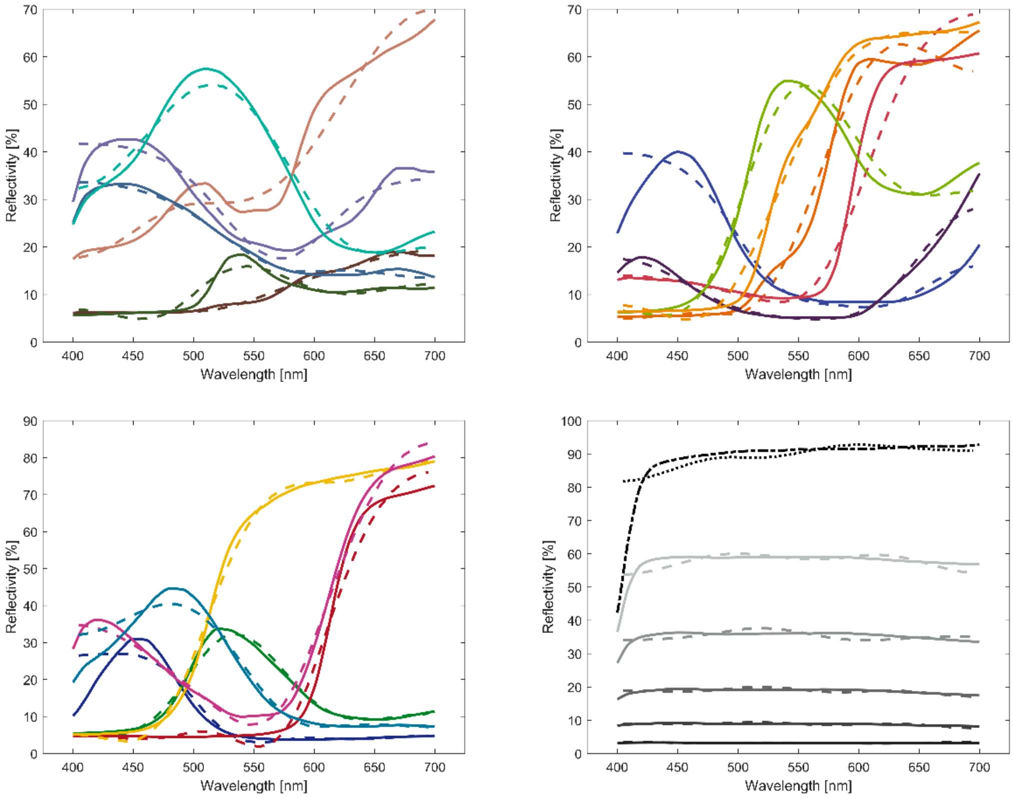


*Figure 5: Reconstructed reflectivity spectra for* $\boldsymbol{T_{min}}$***=1950K****.*

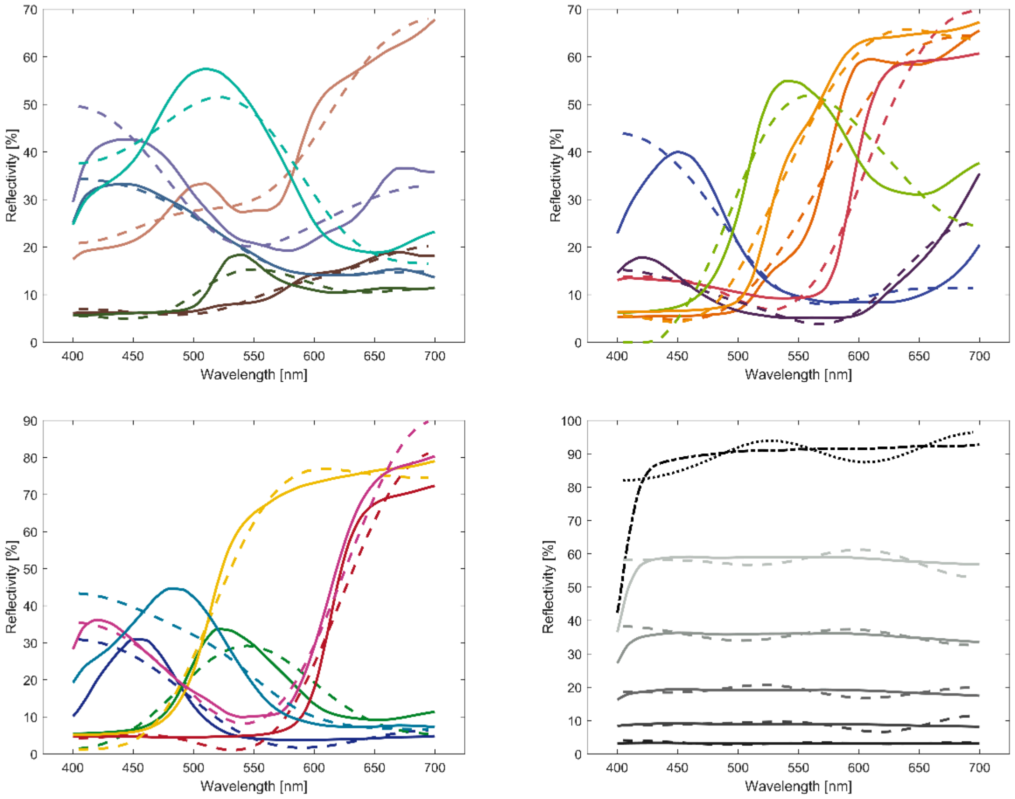


*Figure 6: Reconstructed reflectivity spectra for* $\boldsymbol{T_{max}}$***=2950K**.*

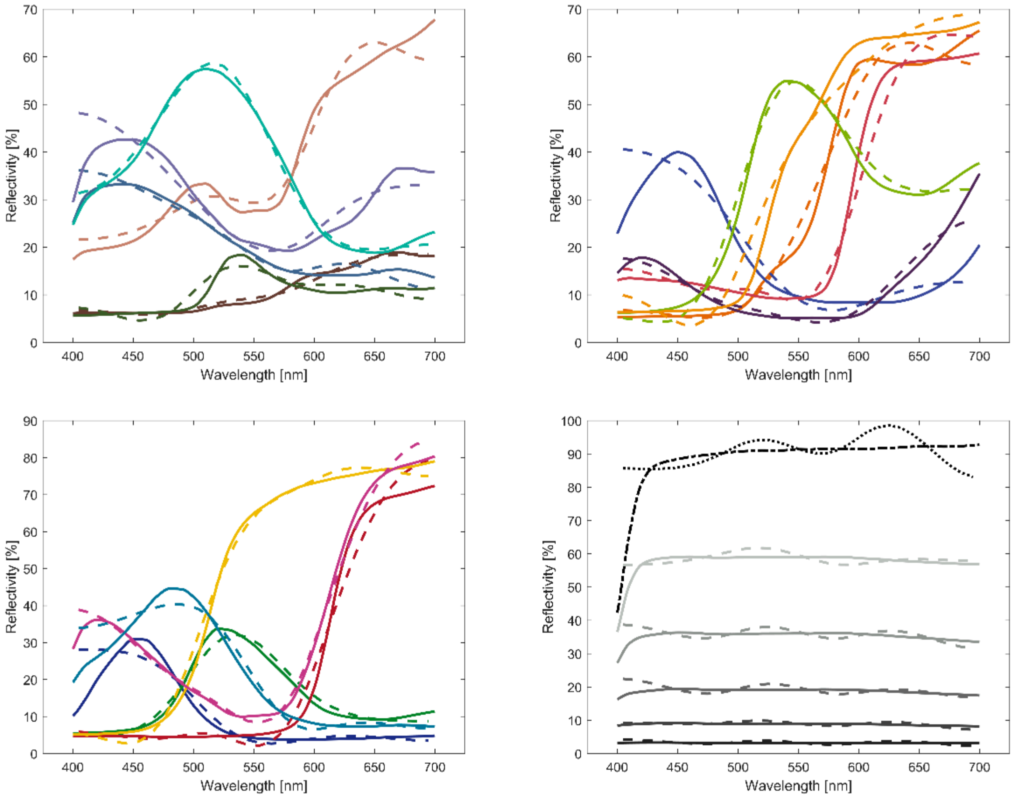


*Figure 7: Reconstructed reflectivity spectra for* $\boldsymbol{\delta T}$***=10K**.*

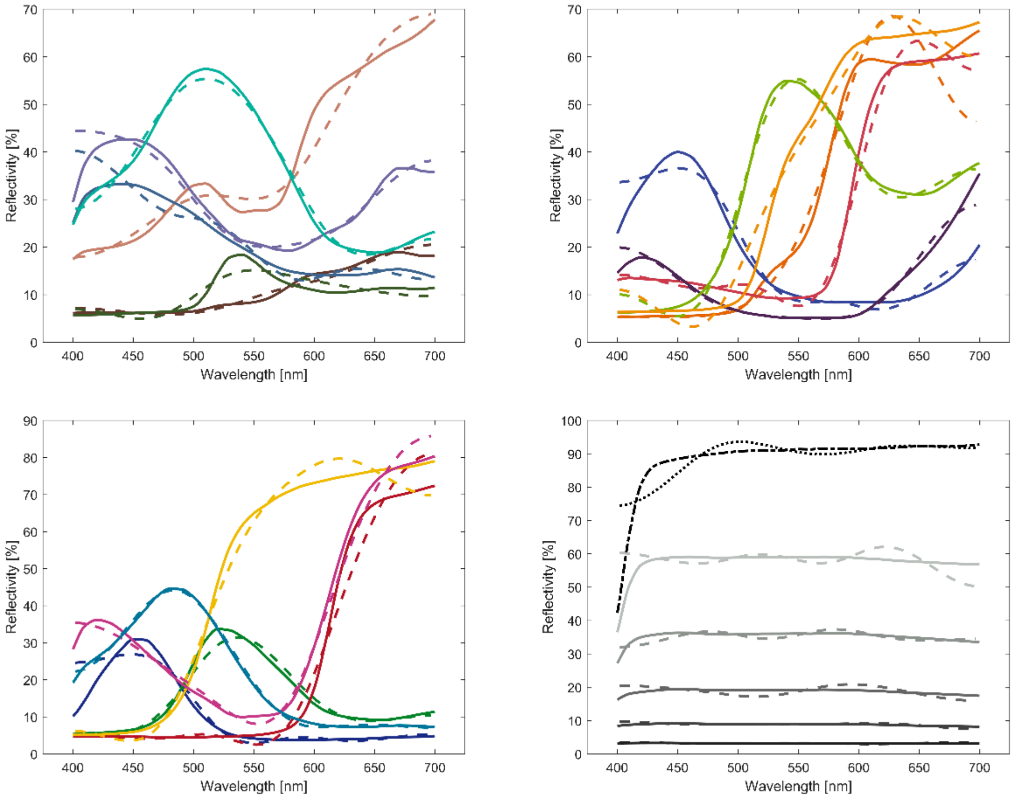


*Figure 8: Reconstructed reflectivity spectra for* ***Δλ=5nm****.*

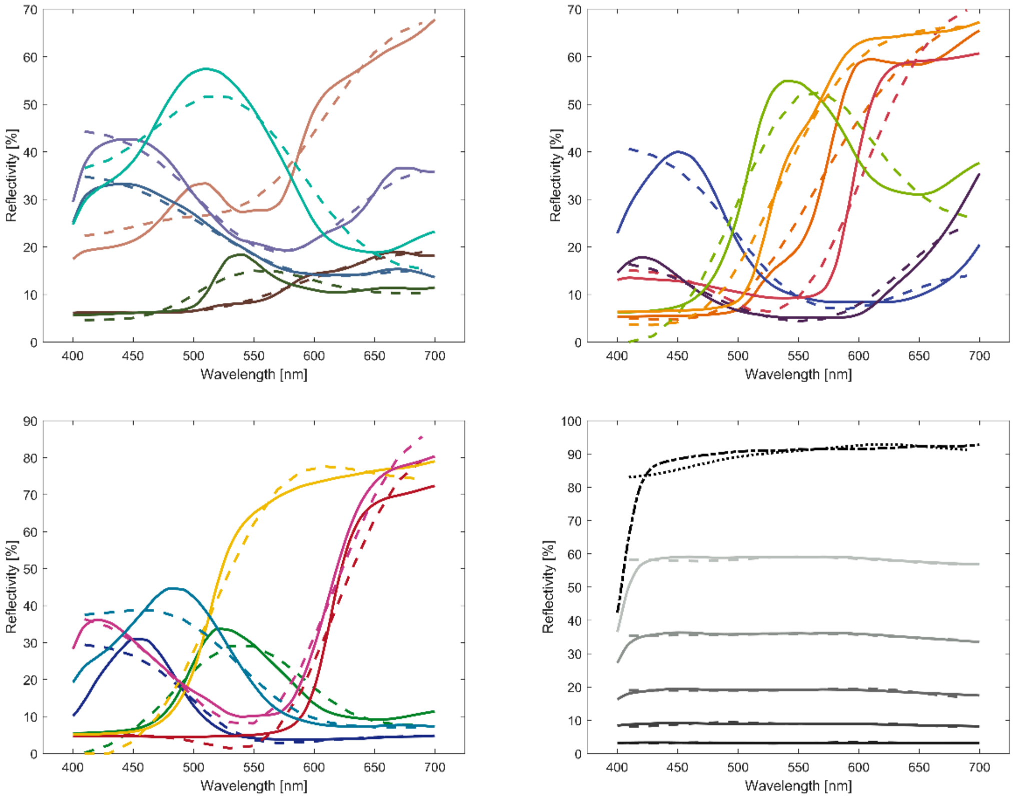


*Figure 9: Reconstructed reflectivity spectra for* ***Δλ=20nm****.*

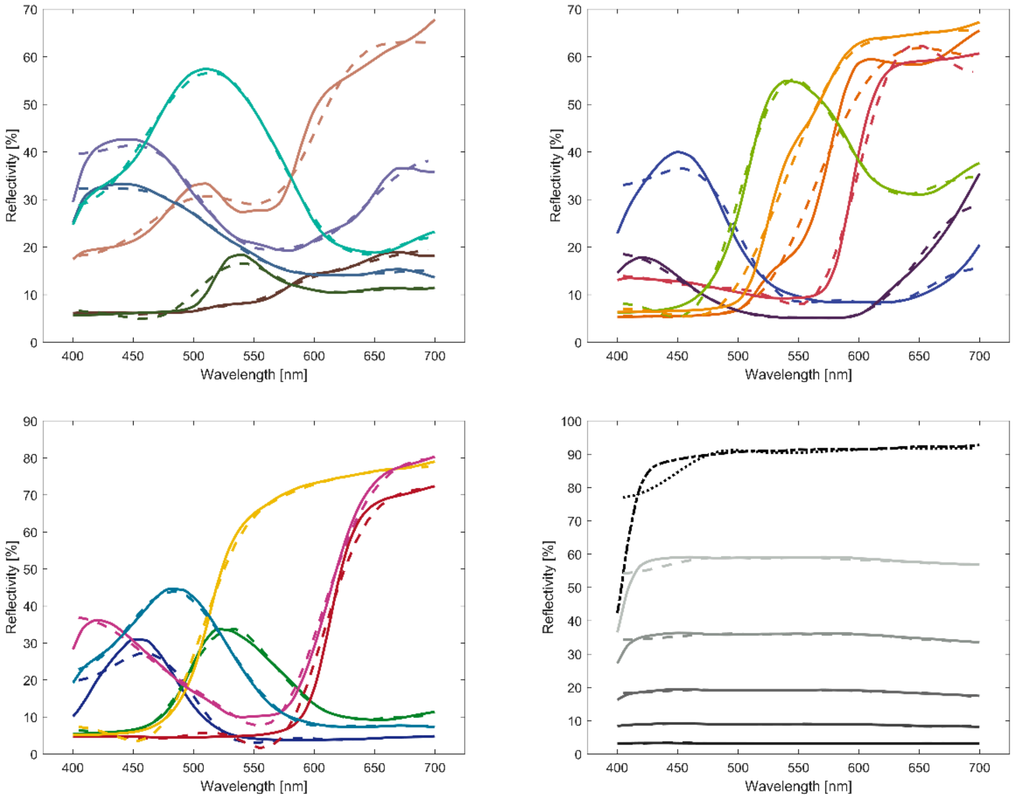


*Figure 10: Reconstructed reflectivity spectra for **N=6** acquisition channels.*
